# Game design elements of serious games in the education of medical and healthcare professions: a mixed-methods systematic review of underlying theories and teaching effectiveness

**DOI:** 10.1007/s10459-024-10327-1

**Published:** 2024-04-02

**Authors:** Alexandra Aster, Matthias Carl Laupichler, Saskia Zimmer, Tobias Raupach

**Affiliations:** Institute of Medical Education, University Hospital Bonn, Venusberg-Campus 1, D-53127 Bonn, Germany

**Keywords:** Serious game, Game design elements, Educational game design, Systematic review, Medical education, Healthcare education

## Abstract

Serious games, as a learning resource, enhance their game character by embedding game design elements that are typically used in entertainment games. Serious games in its entirety have already proven their teaching effectiveness in different educational contexts including medical education. The embedded game design elements play an essential role for a game’s effectiveness and thus they should be selected based on evidence-based theories. For game design elements embedded in serious games used for the education of medical and healthcare professions, an overview of theories for the selection lacks. Additionally, it is still unclear whether and how single game design elements affect the learning effectiveness. Therefore, the main aim of this systematic review is threefold. Firstly, light will be shed on the single game design elements used in serious games in this area. Second, the game design elements’ underlying theories will be worked out, and third, the game design elements’ effectiveness on student learning outcome will be assessed. Two literature searches were conducted in November 2021 and May 2022 in six literature databases with keywords covering the fields of educational game design, serious game, and medical education. Out of 1006 initial records, 91 were included after applying predefined exclusion criteria. Data analysis revealed that the three most common game design elements were points, storyline, and feedback. Only four underlying theories were mentioned, and no study evaluated specific game design elements. Since game design elements should be based on theories to ensure meaningful evaluations, the conceptual GATE framework is introduced, which facilitates the selection of evidence-based game design elements for serious games.

**Supplementary Information:**

The online version contains supplementary material available at 10.1007/s10459-024-10327-1.

## Introduction

Serious games, defined by Michael and Chen (2005) as applications designed not only for entertainment or fun but primarily for having an educational purpose, are currently being used in various educational settings (Cheng et al., 2015). These also include the education of medical and healthcare professionals, where serious games have been shown to increase learning outcome. For instance, the literature describes effective use of a serious game to teach primary care physicians about diabetes mellitus (Diehl et al., 2017) or to enhance a surgeons’ situational awareness (Graafland et al., 2017).

To ensure the teaching effectiveness of a serious game, it should be equipped with game design elements rooted in the construct of gamification. In contrast to full-fledged serious games, gamification basically describes the use and embedment of game design elements in non-game environments (Deterding et al., 2011). Game design elements can be understood as specific components of games (Werbach & Hunter, 2020), that ensure that games are typically defined as games. As Sailer et al. (2013) pointed out, different authors have already attempted to compile lists of game design elements (Kapp, 2012; Robinson & Bellotti, 2013; Werbach & Hunter, 2020; Zichermann & Cunningham, 2011). Besides these lists, Alexiou and Schippers (2018) provided a framework for clustering game design elements into categories of narrative, aesthetics, and core game mechanics, and linked them to aspects of motivational theories. Although several authors have attempted to create useful lists of game design elements, these lists cannot be considered comprehensive.

Cheng et al. (2015) reviewed the foundation of entire serious games on different theories in the broader context of science education (i.e. physics, chemistry, biology, earth science, and interdisciplinary fields). 53% of the studies reported foundations in the theories of constructivism, Vygotsky’s theories, as well as cognitive theories (e.g. cognitive load, flow, and multimedia theory) and activity theory. It is assumed that single game design elements must also be selected based on specific theories in order to be used effectively in serious games. The *Self-Determination Theory* (SDT, Deci & Ryan, 1985; Ryan & Deci, 2000), which originates from the field of motivational psychology, is a theory that is referenced frequently in various fields for the selection of single game design elements (Krath et al., 2021). Satisfying the three basic psychological needs, (i.e. the *needs for autonomy*, *competence*, and *relatedness*) forms the core of the SDT and is supposed to increase intrinsic motivation (Ryan & Deci, 2000). The need for autonomy refers to making independent decisions for possibly meaningful tasks, the need for competence refers to the opportunity of influencing the surrounding environment, and the need for relatedness refers to the inclusion into a relevant social group (Sailer et al., 2017). Those psychological needs can be addressed by different game design elements (Alexiou & Schippers, 2018). Sailer et al. (2017) summarized that the implementation of points, performance graphs, badges, and leaderboards addresses the need for competence. Moreover, distinct and timely feedback addresses this need (Alexiou & Schippers, 2018). Avatars and meaningful stories relate to the need for autonomy (Sailer et al., 2017). In a broader sense, the need for autonomy can be addressed by high levels of perceived control and the given opportunity to express oneself (Alexiou & Schippers, 2018). Meaningful stories and teammates, as well as facilitated interaction between players, refer to the need for social relatedness (Alexiou & Schippers, 2018; Sailer et al., 2017). Taking this into account, the theory-driven selection of game design elements should improve intrinsic motivation and thus learning outcomes. Since the SDT is only one theoretical alternative, other theories like the *Flow theory* or the *Experiential learning theory* can be taken into account for selecting game design elements (Krath et al., 2021). Experiencing the state of flow is defined as being completely immersed in an activity without realizing the passing of time, leading to the activity being intrinsically rewarding as it is conducted for its own sake and not in order to receive an extrinsic reward (Csikszentmihalyi, 1975; Csikszentmihalyi & Larson, 2014).

It is already known that single game design elements can address basic psychological needs that in turn enhance the intrinsic motivation of players. Now the question arises whether the theory-driven selection of game design elements for usage in serious games also applies to the field of education of medical and healthcare professions. Although the effectiveness of a serious game is necessarily affected by different mechanisms, it is of interest whether primary studies assessed the effectiveness of specific game design elements in improving learning outcomes. Since it can be assumed, that the use of game design elements that foster intrinsic motivation may result in increased learning outcomes. The rationale of this review therefore was to gain insight into the used game design elements in serious games for the field of medical education with special attention on their theories and learning effectiveness.

Accordingly, the main aim of this systematic review is threefold divided in the following sequential research questions:

I. Which game design elements are being used in serious games in the education of medical or healthcare professions?
II. What are the theories that the game design elements are based on?
III. How effective are these game design elements in terms of student learning outcome?

## Methods

Compliance to the updated PRISMA statement on systematic reviews (Page et al., 2021) was ensured, and the systematic review was preregistered at Prospero (ID CRD42022333081).

### Search strategy

To identify relevant keywords for the final search strategy, an unstructured search was conducted and a couple of those found serious game studies were screened. Appropriate keywords were gathered and compiled, resulting in three overarching themes covering game design, serious game, and medical education.

A first search was conducted in mid-November 2021 in the following six literature databases: PubMed (National Library of Medicine), ScienceDirect (Elsevier), IEEE Xplore (IEEE), Web of Science (Clarivate), Wiley Online Library (Wiley), and PsycINFO (American Psychological Association). The search strategy comprised keywords from the three areas above in the following composition: (educational game design OR educational design OR game design OR design element*) AND (serious game* OR game based learning OR gamified learning) AND (medical education OR medical student*). The linking of the three domains by the Boolean operator AND was intended to ensure that the studies specifically address game design elements in serious games in medical education. Due to the focus on game design elements embedded in serious games, the term gamification was not explicitly used as this would include studies adding game design elements to other non-game learning contexts. This search strategy was applied to all databases, searching in all fields without filtering for single fields like abstract or title. Furthermore, no filters or limits like year, language, or full availability, were applied. To guarantee topicality and to include papers published in the meantime, another updating search was conducted in the beginning of May 2022 using the same search strategy in the same databases.

### Eligibility criteria

Peer-reviewed primary studies in the field of medical education using a serious game (regardless of being analogue or digital) were included. Medical education concerned under- and postgraduate-level medical students and doctors as well as other healthcare professions, such as nurse, physical therapist, pharmacologist, and other included in patient health care. Studies published until 2022 were included without specifying a start date, as the aim was to encompass research on both analogue and digital games comprehensively and gather as many studies as possible.

Therefore, nine exclusion criteria were predefined, see Table 1 for a listing of the exclusion criteria including respective reasoning. After rating the full text of the first five records, it became apparent, that two more exclusion criteria were needed as the eligibility was not always discernible based on the abstracts. This is illustrated exemplarily for exclusion criterion eight. The eighth exclusion criterion was added after it became apparent that the decision as to whether the game was a full-fledged serious game or merely gamification was sometimes ambiguous when made solely based on the abstract. Thus, criterion eight was applied when records using gamification were included based on the abstract and then excluded based on full text screening. This procedure guaranteed that studies that only dealt with gamification of non-game learning contexts were excluded.

**Table 1:** Exclusion criteria

Number	Label	Explanation
1	no gamification	reports did not consider gamification, or focused on a completely different topic
2	games for public health, patient health or patient education	reports considered education, prevention or treatment in patient contexts, e.g. mental health, brain injury, diabetes, and autism, among others
3	other target groups than medical or healthcare professions education	reports focused on target groups except for medical or healthcare professions education, or patient contexts
4	target group younger than 18 years old	reports considered a target group younger than 18 years old
5	games not created solely for educational purposes	reports used games that were not created solely for educational purposes (e.g. commercial games, gamified learning platforms such as Kahoot! )
6	clear focus on other learning contexts	reports considered gamification or serious games in other learning contexts such as learning disabilities
7	foreign languages	reports published in other languages than English or German
8	obviously, no use of a serious game	reports did not use a serious game but gamification
9	not a primary study	reports were not primary studies but secondary literature (e.g. reviews)

Reasons 8 and 9 were added for the content analysis after screening the full texts of the first records

### Screening strategy

For gathering and organizing the records, Rayyan (Ouzzani et al., 2016; rayyan.ai), a tool that can be used to manage collaboration during a systematic review, was employed. As a first step, all references were imported to Rayyan. Rayyan automatically detected duplicates, which the authors (AA and MCL) checked manually afterwards. Two authors (AA and MCL) performed a blinded assessment of the remaining records based on title and abstract, independently applying the exclusion criteria one to seven. Subsequently, the blind mode was discarded, ratings were compared and the two authors (AA and MCL) systematically resolved disagreements. Whenever necessary the third author (SZ) mediated the discussion. Cohen’s kappa was chosen as an indicator for interrater reliability as it allows for direct interpretation of the joint agreements with excluding agreement by chance (Cohen, 1960). After rating the abstracts, a Cohen’s kappa of κ = 0.97 was achieved. All included and retrievable records were downloaded and made available to all authors. In the following step, the authors (AA and MCL) read all publications, reviewed them for their eligibility based on the full text, and filled in all applicable fields of the data-charting table, independently and blinded again. At this stage, exclusion criteria eight and nine were added, as already mentioned above (refer to Table 1). The content analysis of full texts was conducted with the help of a detailed data-charting table including general information about bibliography of the record (i.e. year of publication, nationality of first author, journal and type of publication, and keywords). In addition, information about the used serious game (i.e. participants, medical field) and information on specific game design elements (i.e. used game design element, proposed theories, evaluation in terms of perception, teaching effectiveness including description and results of the conducted study) were collected. Furthermore, study quality was assessed via the *Medical Education Research Study Quality Instrument* (MERSQI, Reed et al., 2007). MERSQI can be applied for measuring the methodological quality of observational, quasi-experimental, and experimental studies in medical education (Reed et al., 2007). The achievable score can range between 5 and 18.

All information had to be gathered without prioritizing and without collecting any additional data. Results for the third research question (evaluation and effectiveness), were specified according to Kirkpatrick’s four-level model, which consists of the levels reactions, learning, behavior, and results to assess learning outcomes (Praslova, 2010). The evaluation model by Kirkpatrick was used, as it already proved to be an helpful tool for evaluating training outcomes (Smidt et al., 2009). Following the first round of ratings by two authors (AA and MCL), interrater reliability according to Cohen’s kappa was κ = 0.46. After discussing disagreements Cohen’s kappa improved to κ = 0.89. The third author (SZ) moderated the discussion about the remaining unresolved conflicts until an agreement between the authors (AA and MCL) who read the full texts was reached. According to Landis and Koch (1977) the achieved interrater reliabilities of 0.46 and 0.89 can be interpreted as moderate and almost perfect agreements.

Since this review covered a heterogeneous literature landscape, especially in terms of the respective methodologies, no meta-analysis was conducted. Therefore, no statistical values were collected. In case a record presented relevant statistical measurement data, those results were verbalized. Quantitative as well as qualitative records were included. Nevertheless, some aspects of the primary studies such as number of used game design elements were collected quantitative, while other aspects such as theories were recorded qualitatively resulting in a mixed-methods analysis.

### Data synthesis

All data was synthesized narratively, apart from the numerical values recorded with the MERSQI.

In the absence of a universal, comprehensive list of game design elements, the authors specified a predefined list of game design elements of which each was mapped to the categories of Alexiou and Schippers (2018) framework (Table 2). This framework was chosen to sort the game design elements in a meaningful way. The decision to utilize a self-created, predefined list was made to structure and objectify the delineation of game design elements. In seeking a comprehensive list, the decision was made to compile game design elements already documented in the literature. For answering the first research question, the frequency of every single game design element was calculated. This was done first for the total study sample and second for subgroups formed based on the study population (i.e. medical education, and education of healthcare professions). To answer the second and third research question, studies that clearly mentioned theories and tests of effectiveness for game design elements were filtered out.

**Table 2:** Assignment of game design elements to categories

Categories proposed by Alexiou and Schippers (2018)	Authors’ predefined list of game design elements	Explanations
Aesthetics	/	
Narrative	Avatar	Visual player representation (Werbach & Hunter, 2012)
	Storyline	Narrative context of the game (Sailer et al., 2017)
	Connection to real life	Utilization of contents out of real life
	Feedback	Can be provided in numerical, visual, or verbal form
Game mechanics	Points	Numerical feedback that reward or penalize actions of players (Sailer et al., 2017)
	Badges	Form of visual feedback
	Leaderboard	Representation and ranking of individual or group scores
	Rewards	Form of visual feedback
	Visualization of progress	Representation of players progress without ranking
	Collaboration	Teamwork
	Competition	Competing against each other individually or in groups
	Time limit	Time restriction on gameplay
	Additional life	Receiving additional life, e.g. as a reward
	Levels	Different levels of varying difficulty
	Hints / Tips	Support during game play
	Dynamic difficulty	Corresponding to levels

Due to the lack of uniform definitions for all game elements, only some explanations are derived from existing literature

### Assessment of bias

Two authors assessed blinded and independently bias for each record, according to the risk of bias assessment proposed by the Joanna Briggs Institute by means of the respective applicable Critical Appraisal Tools. Due to the different types of studies, different assessment tools were used. Specifically, the Critical Appraisal Tools for Analytical Cross-sectional Studies (Joanna Briggs Institute, 2020a), Qualitative Research (Joanna Briggs Institute, 2020b), Quasi-experimental Studies (Joanna Briggs Institute, 2020c), and Randomized Controlled Trials (Joanna Briggs Institute, 2020d) were used.

## Results

### Study selection

**Fig. 1:**
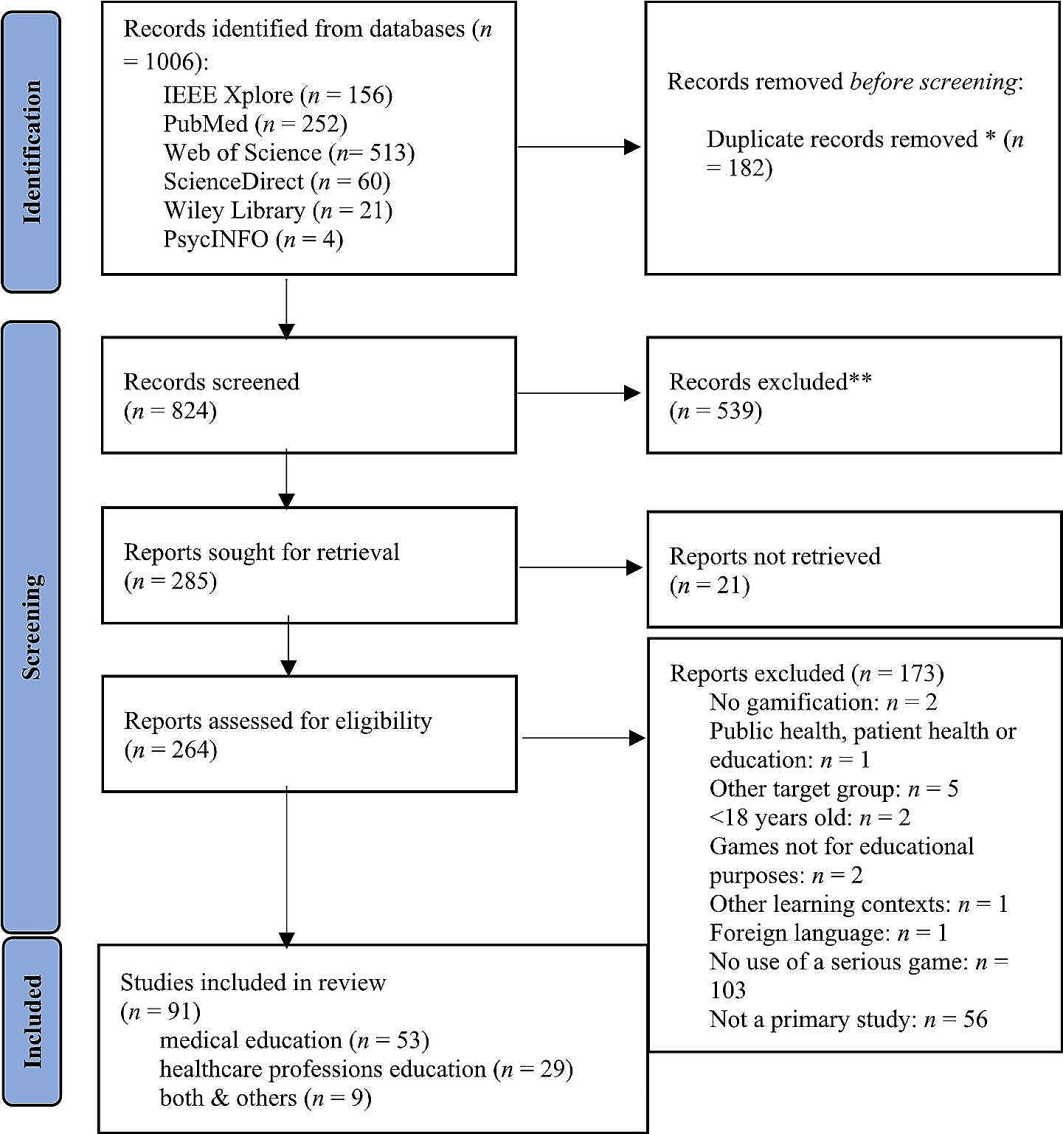
PRISMA flow chart. * Duplicates were detected automatically by Rayyan (rayyan.ai), nevertheless the executive decision on whether it was a duplicate was made by the authors. ** At this stage, all reports were excluded manually by the authors without the help of an automation tool

The database searches initially yielded 1006 results of which 182 (18%) were duplicates. The remaining 824 (82%) abstracts were screened for eligibility, which lead to the exclusion of 539 (54%) records, resulting in 285 (28%) records for full text screening. Since 21 records were not retrievable, a total number of 264 (26%) records were screened for content analysis. In total, 173 (17%) records were excluded with the vast majority resulting from exclusion reason eight (103, 60%). The frequent occurrence of exclusion reason eight is attributable to inconsistent use of the term “serious game”. It is not always used for true serious games as defined by Michael and Chen (2005), but also for simulations, gamified scenarios, or for the use of gamified platforms or commercial games (e.g. Burns et al., 2021; Ismail et al., 2019; Turley et al., 2007). For further information on the search and selection process as well as on the distribution of exclusion reasons, please refer to the PRISMA flow chart in Fig. 1. Conclusively, 91 (9%) records were included.

### Study characteristics

The included studies can be categorized according to the broader educational field from which the study populations were drawn and according to the respective subgroups. More than half of all included studies (*n* = 53; 58%) covered medical education, 29 (32%) referred to other healthcare professions, and 9 (10%) addressed both or other related fields (see Table S1 in the supplementary material).

Studies conducted in the field of medical education can be further specified by categorizing the subgroups, mainly medical students were studied (Agudelo-Londono et al., 2019; Alyami et al., 2019; Anyanwu, 2014; Asadipour et al., 2015; Backhouse & Malik, 2019; Boeker et al., 2013; Borro Escribano et al., 2015; Chang et al., 2021; Dankbaar et al., 2016; Dankbaar, Richters, Dankbaar et al., 2017a, b; De la Cruz et al., 2018; Donovan et al., 2021; Drummond et al., 2017; Faber et al., 2018; Gauthier et al., 2015; Hannig et al., 2012;; Hu et al., 2021b, c; Janssen et al., 2015; Kanthan & Senger, 2011; Karbownik et al., 2016; Katrikh et al., 2021; Kinio et al., 2019; Lagro et al., 2014; Lopez Chavez et al., 2020; Mlika et al., 2020; O’Leary et al., 2005; Palee et al., 2020; Qin et al., 2009; Ribeiro et al., 2013; Schmidt & Grigull, 2017; Sward et al., 2008; Tan et al., 2022; Tsopra et al., 2020; Zielke et al., 2016). Additionally to the approach of solely evaluating medical students, some studies combined medical students with residents or physicians in their population (Diehl et al., 2015a, b; Graafland et al., 2015; Hale et al., 2021; Kaul et al., 2021; Nemirovsky et al., 2021; Rodrigues et al., 2020). Other studies solely focused on trained residents or physicians (Boulet et al., 2007; Dankbaar, Roozeboom et al., 2017; Diehl et al., 2013; Diehl et al., 2017; Graafland et al., 2017; Katz et al., 2017; Mohan et al., 2017; Mohan et al., 2018; Silverio & Chen, 2019; Telner et al., 2010; Ward et al., 2019).

The second largest group of studies, after medical students, addressed education of healthcare professions other than medicine. Many of these studies included nurses and nursing students (Barr et al., 2008; Bonet et al., 2021; Calik et al., 2022; Chang et al., 2019; Hu et al., 2021a; Johnsen et al., 2016a, b, 2018, 2021; Merilampi et al., 2021; Su, 2016; Tan et al., 2017). Other studies frequently addressed populations such as pharmacy students, pharmacists and pharmacy technicians (Cole & Ruble, 2021; Cusick, 2016; Shi et al., 2020), dental or dental surgery students (Aubeux et al., 2020; Wu et al., 2021), as well as physiotherapists (Savazzi et al., 2018) or physiotherapy students (Ferrer-Sargues et al., 2021). Further studies focused on nursing and paramedical students (Saeidmirzaei et al., 2020), occupational therapy students (Dugnol-Menendez et al., 2021), pharmacy and nursing (Kayyali et al., 2021), operating room technology students (Akbari et al., 2022), paramedic students (Aksoy, 2019), health polytechnics students (Sunindya & Purwani, 2017), advanced life support providers (Buttussi et al., 2013), health advisors (Basole et al., 2013), and interprofessional students (Friedrich et al., 2019). Oliveira et al. (2021) did not specify their study population.

Some studies addressed populations from both educational fields of medical and healthcare professions (Abensur Vuillaume et al., 2021; Buijs-Spanjers et al., 2020; Donald et al., 2017; El Mawas & Cahier, 2013; Graafland et al., 2014; Jackson et al., 2020; Knight et al., 2010; Sanders et al., 2020; Tsoy et al., 2019).

**Fig. 2:**
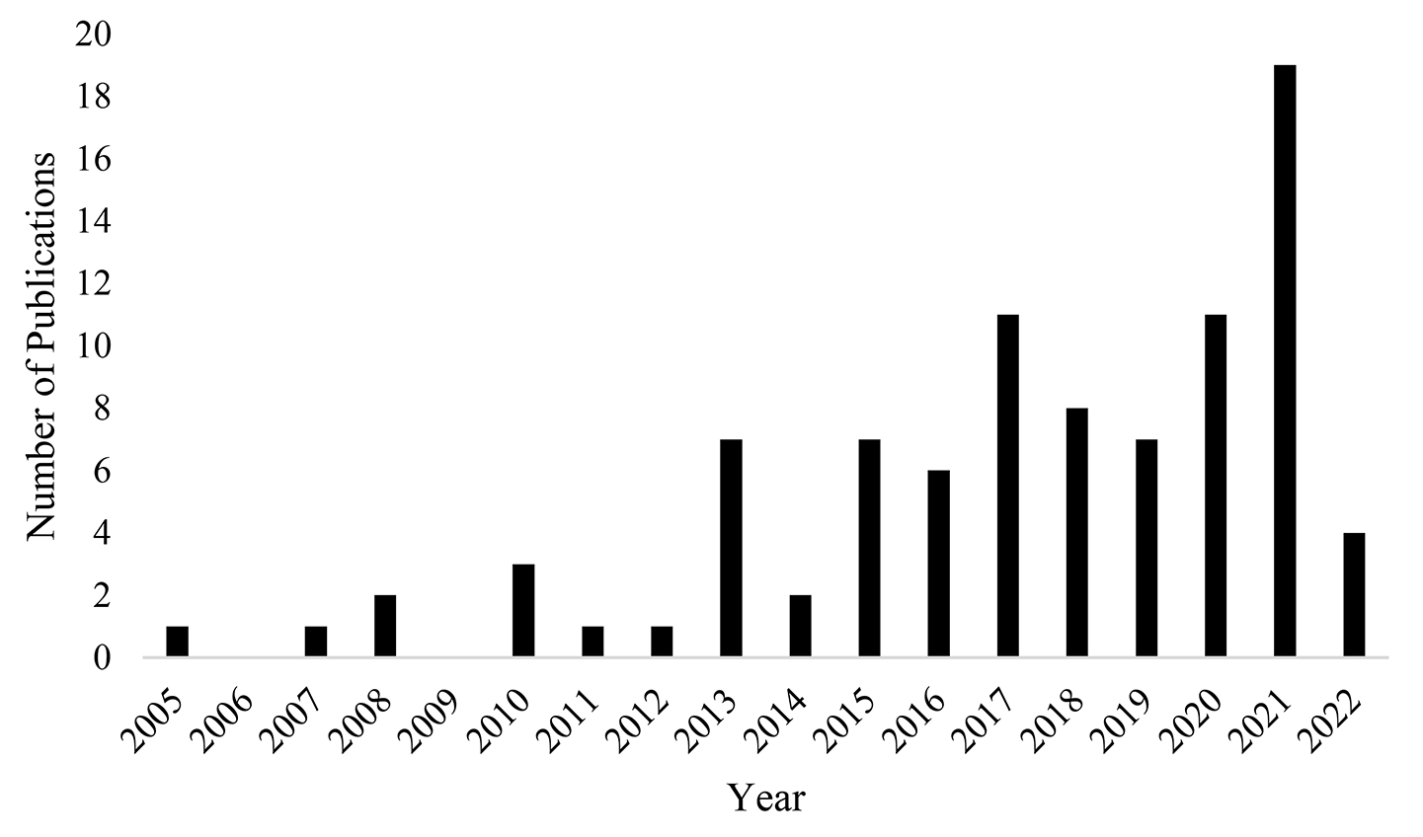
Numbers of publication per year

**Fig. 3:**
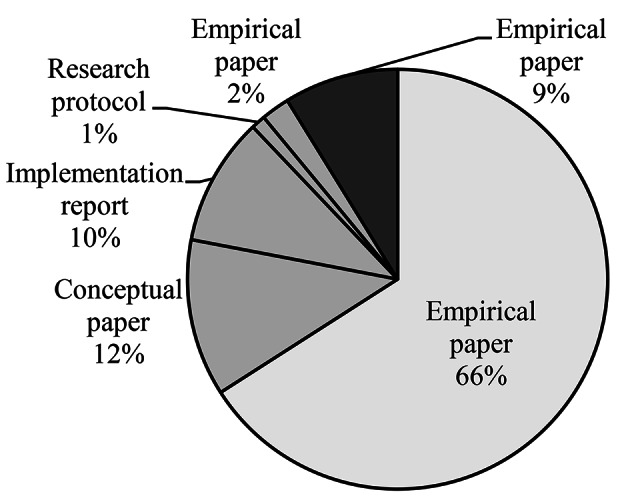
Distribution per study type The area in light grey represents the overarching category “quantitative”. Areas in middle grey represent the overarching category “qualitative”. Areas in dark grey represent the overarching category “mixed-methods”

As shown in Table 3, a majority of studies originated from the USA. Studies were mostly published in 2021 with the first being published in 2005 (refer to Fig. 2). In terms of study type, quantitative empirical papers formed the largest category. The remaining records reported qualitative studies (i.e. conceptual paper, implementation record, research protocol, empirical paper) and results of mixed-methods empirical research (refer to Fig. 3).

**Table 3:** Study characteristics across all populations (*N* = 91)

Characteristics	Absolute number	Relative number
Country of origin		
USA	18	(20%)
Netherlands	9	(10%)
Canada	7	(8%)
UK	6	(7%)
Brazil, China, France,	Each 5	(5%)
Norway, Spain	Each 4	(4%)
Germany, Taiwan	Each 3	(3%)
Iran, Italy, Singapore, Turkey	Each 2	(2%)
Australia, Colombia, Finland, Indonesia, Ireland, Mexico, New Zealand, Nigeria, Peru, Poland, Portugal, Switzerland, Thailand, Tunisia	Each 1	(1%)
Modality of the game		
digital	69	(76%)
analogue	21	(23%)
hybrid	1	(1%)

All relative frequencies were calculated based on the total number of included studies and were rounded to the nearest integer

### Risk of bias assessment and quality of studies

The methodological quality, as assessed by the MERSQI rating, reached a mean rating of *M* = 11.3 with a Kendall rank correlation coefficient of τ = 0.384 between the two raters. This effect size can be interpreted according to Cohen (1988) as a medium coherence.

According to the Critical Appraisal Tools by the Joanna Briggs Institute, studies are categorized into having a low, moderate, or high risk of bias. The risk of bias assessment was applicable for 67 studies, of which the majority (*n* = 47) was classified as having high risk of bias. Only a minority of studies were classified as having a moderate (*n* = 14) or low risk of bias (*n* = 6; see Table S1 in the supplementary material).

### Research question 1: design elements in serious games in medical education

All recorded game design elements were mapped on the categories proposed by Alexiou and Schippers (2018) to ensure a concise evaluation. Across all populations, the three most frequently used game design elements in serious games were storyline, points and feedback (Table 4). In studies relating to the education of healthcare professions, serious games were commonly equipped with time limit. Serious games in studies addressing both educational fields of medical and healthcare professions frequently used collaborative elements.

**Table 4:** Absolute and relative frequencies of used game design elements in the respective populations

Categories by Alexiou and Schippers (2018)	Game design element	Total sample (*N* = 91)		Medical education (*n* = 53)		Healthcare professions education (*n* = 29)		Both (*n* = 9)	
Aesthetics	/								
Narrative	Avatar	21	(23%)	14	(26%)	6	(21%)	1	(11%)
	Storyline	48	(53%)	28	(53%)	16	(55%)	5	(55%)
	Connection to real life	2	(2%)	1	(2%)	1	(3%)	0	(0%)
	Feedback	38	(42%)	26	(49%)	7	(24%)	5	(55%)
Game mechanics	Points	45	(49%)	31	(58%)	12	(41%)	2	(22%)
	Badges	7	(8%)	5	(9%)	2	(7%)	0	(0%)
	Leaderboard	10	(11%)	8	(15%)	3	(10%)	1	(11%)
	Rewards	13	(14%)	11	(21%)	2	(7%)	0	(0%)
	Visualization of progress	6	(7%)	5	(9%)	1	(3%)	0	(0%)
	Collaboration	24	(26%)	14	(26%)	7	(24%)	3	(33%)
	Competition	14	(15%)	11	(21%)	2	(7%)	2	(22%)
	Time limit	26	(29%)	15	(28%)	9	(31%)	2	(22%)
	Additional life	5	(5%)	5	(9%)	0	(0%)	0	(0%)
	Levels	16	(18%)	12	(23%)	4	(14%)	1	(11%)
	Hints / Tips	21	(23%)	15	(28%)	5	(17%)	1	(11%)
	Dynamic difficulty	13	(14%)	9	(17%)	3	(10%)	2	(22%)

All relative frequencies were calculated based on the total number of included studies in the respective population category were rounded to the nearest integer

Two reviewers listed each present game design element of the respective studies. The consensus among the reviewers in recognizing each game design element was described using the Kendall rank correlation coefficient, resulting in τ = 0.514 for game design elements in the total study sample, τ = 0.397 especially for medical education, τ = 0.631 for the education of healthcare professions, and τ = 0.562 for studies examining both populations. Thus, the consensus regarding the total sample, the sample regarding the education of healthcare professions, and the sample for both populations can be interpreted as strong, whereas the coherence for the medical education sample was medium (Cohen, 1988).

### Research question 2: underlying theories

A minority of studies mentioned underlying theories for the design or construction phase of the entire serious game. Even less studies, numerically four studies, mentioned underlying theories for the selection of integrated game design elements. Two of these belonged to the field of medical education whereas the other two belonged to the education of healthcare professions.

Tan et al. (2022) referred to the Self-Determination Theory by Deci and Ryan (1985) not only for the entire game development, but also for the selection of specific game design elements. The authors assigned the selection of game design elements to the theory’s main components (Tan et al., 2022). To fulfill the need for competence, participants could refer to already acquired knowledge as the game materials referred to completed modules. Furthermore, participants were allowed to gather additional information for answering the proposed questions in the serious game. The use of fewer rules and minimal restrictions helped to fulfill the need for autonomy. Lastly, the collaboration in teams of two players fulfilled the need for relatedness.

Mohan et al. (2018) followed the theories of narrative engagement and analogical reasoning. The authors named both theories as the foundation for the use of a storyline (Mohan et al., 2018). Following the theory of narrative engagement, an integrated storyline helps to promote decision-making competences in medical students, which can be transferred to related situations. Besides, the theory of analogical reasoning assumed that structured case comparisons are effective in training the mastery and application of decision-making principles. Additionally, this theory served as the basis for the selection of the puzzle character for the serious game.

Shi et al. (2020) referenced the development of the serious game and the selection of its integrated game design elements, especially the embedment of a storyline, to the RETAIN model. The authors described the RETAIN model as consisting of the elements “relevance, embedding, transfer, adaptation, immersion and naturalization” (p. 48).

Kayyali et al. (2021) based the selection of game design elements on the four player types defined by Bartle (1996), i.e. killer, achiever, explorer, and socializer. Accordingly, the embedment of achievements like medals, titles, or ranks, and a public leaderboard including the respective scores, address the types of killers and achievers. In other words, the “PBL triad” consisting of ‘points, badges, and leaderboard’, which is frequently mentioned in the literature as characteristic game design elements (Werbach et al., 2012), can appeal to these player types. Kayyali et al. (2021) further state that leaderboards as well as interactive online functions motivate the socializer, while the usage of narratives appeals to the explorer.

### Research question 3: teaching effectiveness of game design elements

Among a subset of 76 studies which were suitable for a classification according to Kirckpatrick’s four-level training evaluation model, a majority (*n* = 39) evaluated the interventions on the second level (learning). Another large number of studies only assessed student reactions (*n* = 27). While none of the covered studies evaluated their serious game on the highest level of Kirkpatrick’s model (i.e. results), 10 studies assessed outcomes on the level of behavior. Studies that exclusively examined students’ reactions cannot contribute to the evaluation of the teaching effectiveness. Since the third research question focuses on the effectiveness of specific game design elements, studies that solely assessed learning with the entire serious game cannot be consulted.

To provide a valuable assessment of teaching effectiveness, it is desirable to have a theoretical foundation. None of the studies, which based the selection of game design elements on a theoretical foundation, tested the teaching effectiveness of specific game design elements.

### Conceptual GATE framework

**Fig. 4:**
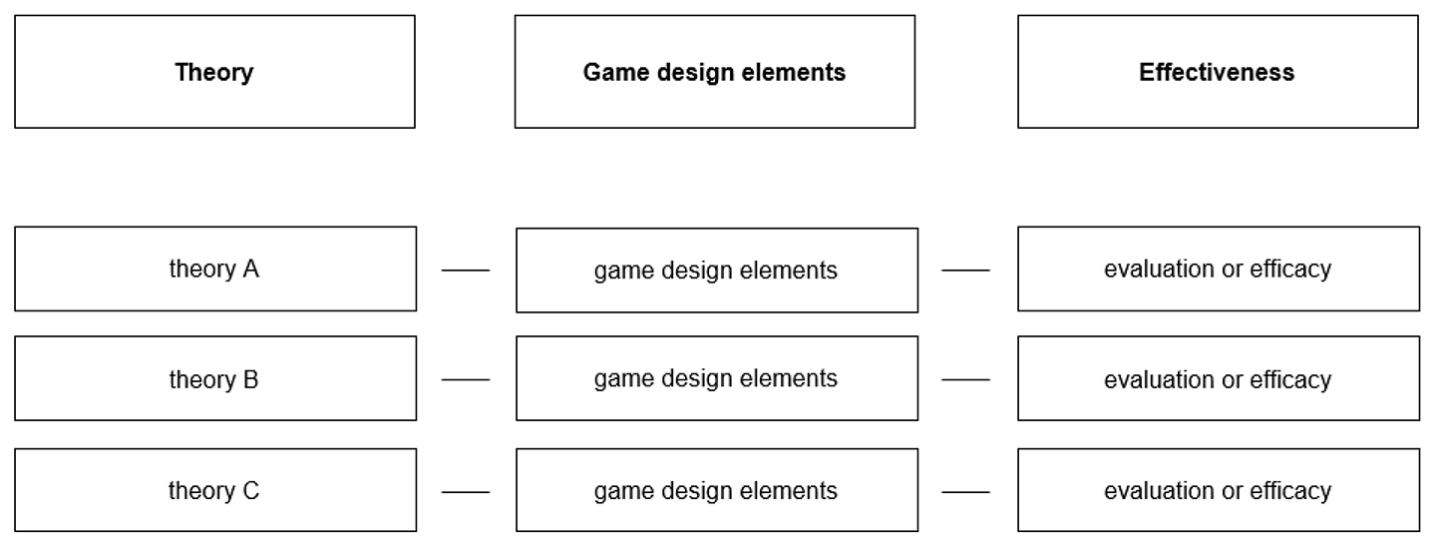
Schematic representation of a template for the GATE framework

The threefold approach of this review suggested the need to develop a conceptual framework for the theory-oriented selection of game design elements that combines the three categories: theory, game design elements, and effectiveness (schematically depicted in Fig. 4). In this context, game design elements are subsumed under the respective theories with the theories serving as the foundation for the respective game design elements. The goal of this conceptual framework is to provide guidance to researchers for selecting evidence-based and avoiding redundant or inappropriate game design elements. Besides the use during the design phase, the framework may also be an useful tool for the evaluation of game design elements.

Table 5 presents an application of the framework to the findings of this review. This was achieved by incorporating all theories identified for Research Question 2 into the framework (Kayyali et al., 2021; Mohan et al., 2018; Shi et al., 2020; Tan et al., 2022). The superscript numbers as well as the notes of the table, refer to the respective primary study. As a result, the current conceptual framework comprises four theories underlying the game design elements. Additionally, since these theories also encompassed subcategories upon which the game design elements are grounded, the category “subcategories” was further added. The domain of game design elements was supplemented with two additional game design elements (i.e. online functions for players and rules) that had been identified during the analysis of the included studies. In general, the majority of studies did not provide clear connections between game design elements, theories, and outcomes. Thus, this review only found a small number of studies that mentioned theoretical foundations of which none applied appropriate evaluation or effectiveness testing to date. As far as the authors are aware, this combination represents a novel approach. Therefore, it should serve as a focus for future studies seeking to delve deeper into this intersection. It could be assumed that in the future, only theories and corresponding game design elements accompanied by an effectiveness assessment will be included in the framework. Nevertheless, it already comprises the theoretical basis for some game design elements.

**Table 5:** GATE framework consisting of the triad of game design elements, related theories and effectiveness assessment

Theory	Subcategories	Game design element	Effectiveness
SDT ^1^	Need for competence	Connection to real life (e.g. in terms of use of educational material)	/
	Need for competence	Hints / Tips (e.g. in terms of using educational material)	/
	Need for relatedness	Collaboration	/
	Need for autonomy	Rules (e.g. in terms of minimal rules)	/
Player types by Bartle	Killer & Achiever	Points	/
	Killer & Achiever	Badges	/
	Killer & Achiever & Socializer	Leaderboard	/
	Socializer	Online functions for players	/
	Explorer	Storyline	/
Narrative engagement & analogical reasoning ^2^		Storyline	/
RETAIN model ^3^		Storyline	/

Explorer, Killer, Achiever, and Socializer refer to the player type theory by Bartle (1996) as worked out by Kayyali et al. (2021). The examples given for the game design elements originate from the respective primary studies

^1^ Deci and Ryan (1985) found in Tan et al. (2022), ^2^ Mohan et al. (2018), ^3^ Shi et al. (2020)

## Discussion

### General discussion

This systematic review attempts to take a threefold look at the use of game design elements in serious games in the education of medical and healthcare professions. First, it was of interest which game design elements were generally used. Second and third, it was of interest whether the selection of game design elements was based on established theories and whether their effectiveness in terms of student learning outcome were evaluated. Overall, 91 studies met the inclusion criteria and were analyzed for answering the three aims of the review.

Across all populations, the most frequently used game design elements were storyline, points, and feedback. Points can be understood as basic game design elements that reward or penalize the actions of players and thus function as a numerical progress representation, and are closely associated with feedback (Sailer et al., 2017). The implementation of feedback, especially immediate feedback, is essential for motivating players, as it can act as negative or positive reinforcement (Sailer et al., 2013). Feedback can be provided in different forms, for example in visual form via badges or rewards, numerically via points and scores, or verbally via text-based procedural feedback. Storylines are detached from performance but are relevant for immersion into the serious game as well as for motivating the player by embedding the game activities into a narrative context (Sailer et al., 2017). In general, the majority of game design elements fell within the category of game mechanics. Game mechanics contribute to the cognitive skills und cognitive engagement of the player (Alexiou & Schippers, 2018). This leads to the assumption that game design elements falling within this category are more often used in the field of medical education as this is a subject that has high learning demands. This assumption raises the question of whether a serious game with inherent game design elements from the category game mechanics improves learning outcomes compared to, for example, a serious game with inherent game design elements from the category narrative. Furthermore, it is assumed that narrative game design elements foster empathy as they allow for an identification with the overarching storyline or game characters, thereby facilitates learning on the model (Alexiou & Schippers, 2018). It should also be investigated whether this effect also applies to the field of medical education. One aim of this review was to provide an overview which game design elements are generally used in serious games in the educational field of medical and healthcare professions. Further studies could aim for investigating the effects of certain combinations of game design elements.

A minority of included studies based the selection of game design elements on established theories. Focus was placed only on studies in which theories were specifically mentioned. Otherwise, it would not be possible to say with certainty if and which theories were used. All four studies mentioning a theory opted for different ones. Although the Self-Determination Theory is a frequently referenced theory when it comes to gamification, it was only mentioned once in the field of medical education (Tan et al., 2022). The authors assigned game design elements of their serious game to the particular components of the theory. Thus, the abandonment of stringent rules resulting in minimal restrictions was related to the fulfillment of the need for autonomy (Tan et al., 2022). The fact that the materials embedded into the game originated from finished modules and participants were allowed to seek answers in further materials, was associated to facilitating the need for competence (Tan et al., 2022). The third main component of the Self-Determination Theory, i.e. need for relatedness, was assumed to be fulfilled by the collaboration in two-person teams (Tan et al., 2022). The use of the specific game design element storyline was, on the one hand, based on the theories of narrative engagement and analogical reasoning (Mohan et al., 2018) and, on the other hand, on the RETAIN model containing the elements of “relevance, embedding, transfer, adaptation, immersion and naturalization” (Shi et al., 2020, p. 48). It should be noted that the game design elements for which underlying theories were mentioned do not correspond to the three most commonly used game design elements.

A different approach to the selection of game design elements was found in the report by Kayyali et al. (2021) in which game design elements were selected based on the player types defined by Bartle (1996). It is questionable whether a serious game including game design elements based on different player types can be suitably applied to a broader audience like medical students. Hence, it should be investigated if it is feasible to determine the player types in large study programs in advance and conclusively develop possible versions of the serious game based thereon. Kayyali et al. (2021) mentioned that the user feedback on their entire serious game revealed the most preferred and least preferred game design elements. Time limit, feedback, and hints built the group of most preferred items, while storyline, time limit, and ranks built the group of least preferred items. Since time limit was the most frequently named game design element in both categories, Kayyali et al. (2021) concluded that its perception depends strongly on the user. Therefore and due to its frequent usage, time limit should be evaluated in further studies.

As described above, the third research question dealt with the assessment of the game design elements’ teaching effectiveness. Neither evaluations nor assessments of teaching effectiveness were conducted in terms of specific game design elements but only in terms of the entire serious game. None of the studies that based their selection of game design elements on established theories also conducted an evaluation or effectiveness testing of specific game design elements. However, using a theoretical framework at the design stage can be helpful to enable useful effectiveness testing of a serious game or specific game design elements (Maheu-Cadotte et al., 2021).

Regarding the quality of the included studies, the mean MERSQI score was in line with the average MERSQI score of a frequently referenced study by Cook and Reed (2015). Based on the Joanna Briggs Institute Critical Appraisal Tools, the vast majority of included studies were categorized as having a high risk of bias. Nevertheless, it has to be kept in mind that a risk of bias assessment was not always applicable due to the study characteristics. Another noteworthy aspect related to the quality of studies was the evaluation level of Kirkpatrick’s four-level training evaluation model. Altogether, the Kirkpatrick rating was used for the vast majority of the studies, which indicates that most studies were evaluated at the level of learning, followed by the levels of reactions and behavior. Since only a small number of studies reached the behavior level, evaluation in the included studies lead to the assumption of insufficient satisfaction.

### Limitations

The conjunction of the different keyword sections limits the applied search strategy. All three sections were linked with the Boolean operator AND, which could have resulted in wrong negative or missing results, as maybe not all keywords applied for all relevant studies (e.g. exclusion of studies that examined serious games in medical education without specifically considering game design, see Evans et al., 2015; Watsjold & Zhong, 2020). For a detailed overview of the keywords for each study, see Table S1 in the supplementary material. Out of 91 included studies, only four studies reported underlying theories. Nevertheless, the remaining 87 provided information about used game design elements in the broad field of medical education. One could argue that these studies could have been excluded beforehand. However, they provide relevant insight that although studies embed selected game design elements in their serious game, there is no theoretical foundation. Hence, while developing the conceptual GATE framework, the focus was primarily on those studies that reported theories and associated evaluations or assessments of teaching effectiveness. Most of the included studies exhibited a low Kirkpatrick Level, which may stem from the limited application of rigorous study designs to assess learning outcomes. Furthermore, since there is no exhaustive list of game design elements, the elements used for this review were derived beforehand by the authors from a sub-sample of literature. On this account, it has to be argued that answering the first research question is neither exhaustive nor exploratory, but merely a frequency count of predefined game design elements. Although the approach of using a predefined list allows for a structured and objective overview of used game design elements, an open view for game design elements should be applied in further studies. Still, the extraction of information on game design elements by the two authors only showed moderate agreement. The lack of uniform definitions for game design elements may have led to the two authors’ sometimes slightly different assessments of the respective game design elements. However, the framework by Alexiou and Schippers (2018) served as a basis for the discussion and the selection of game design elements searched in the studies. Further studies should evaluate whether the specific game design elements were allocated to the appropriate categories. The lack of information in Table 5 reveals that several studies indeed mentioned game design elements but without naming underlying theories (e.g. avatars were mentioned in 21 studies while none of them named an underlying theory as a basis). Even more striking, several studies mentioned game design elements but did not evaluate or test the teaching effectiveness of individual game design elements (e.g. Donald et al., 2017; Faber et al., 2018; Nemirovsky et al., 2021). It must be considered that the above findings are based on studies in the broader context of medical education and not on studies of serious games in other educational contexts, which may provide a more precise theoretical background.

### Conclusion

This systematic review, covering the use of game design elements in serious games in the education of medical and healthcare professions, demonstrated that only a minority of game design elements have a theoretical foundation. Moreover, a reliable and valid assessment of teaching effectiveness is missing in the majority of studies, especially regarding the teaching effectiveness of specific game design elements. Based on the heterogeneous findings in the literature landscape, it cannot be conclusively determined whether serious games in general or their inherent game design elements in particular provide an educational benefit. For the development and reporting of further studies, it is suggested to base the selection of specific game design elements on well-established theories as well as to use adequate methodological tools for the assessment of teaching effectiveness. In this regard, the conceptual GATE framework may help select evidence-based game design elements.

## Electronic supplementary material

Below is the link to the electronic supplementary material.

Supplementary Material 1

## Data Availability

The datasets are available from the corresponding author upon reasonable request.
