# Supplementary material for: Game design elements of serious games in the education of medical and healthcare professions: a mixed-methods systematic review of underlying theories and teaching effectiveness
Source: Adv Health Sci Educ Theory Pract. 2024 Apr 2;29(5):1825–48. doi: 10.1007/s10459-024-10327-1 (PMC11549195; doi:10.1007/s10459-024-10327-1)
Supplement: Supplementary file 1 — Supplementary Material 1 [file 10459_2024_10327_MOESM1_ESM.docx]

**Table S1**

Study characteristics grouped by studies examining medical education, healthcare professions education and both populations education

| Title | First author & Year of publication | Keywords | Serious game | Risk of bias assessment |
| --- | --- | --- | --- | --- |
| A comparative case study of 2D, 3D and immersive-virtual-reality applications for healthcare education | Chavez et al. (2020) | healthcare education; virtual reality; comparative case study | MediTool is a VR-based game for studying clinical cases | High risk of bias |
| Adaptive serious game as a learning approach for microbiology | De la Cruz et al. (2018) | bayesian networks; serious games; prediction of student performance | Serious game that teaches medical students selected aspects of microbiology | High risk of bias |
| A game-based training approach to enhance human hand motor learning and control abilities | Asadipour et al. (2015) | / | Adapted from the infinite-runner genre, where one bird has to be lifted up by pressing with index finger different force levels according to palpation pressures. | High risk of bias |
| A little healthy competition: Using mixed methods to pilot a team-based digital game for boosting medical student engagement with anatomy and histology content | Janssen et al. (2015) | / | They Know: Anatomy is a team-based strategy game developed from the platform They Know (which provides the opportunity of creating strategy games for varying educational contexts). | High risk of bias |
| Anatomy adventure: A board game for enhancing understanding of anatomy | Anyanwu (2013) | gross anatomy education; medical education; educational gaming; board games; anatomy reviews; learning styles; educational intervention | The Anatomy Adventure Game is an analogous board game for learning human anatomy, namely nerval, general, vascular, and muscular and osteology anatomy, for second-year students. | High risk of bias |
| An experimental study on the effects of a simulation game on students' clinical cognitive skills and motivation | Dankbaar et al. (2016) | simulation game; fidelity; cognitive skills; motivation; cognitive load | Simulation game (abcdeSIM) to train cognitive emergency care skills by treating an emergency patient case within 15 minutes. | High risk of bias |
| An exploratory digital board game approach to the review and reinforcement of complex medical subjects like anatomical education: Cross-sectional and mixed methods study | Tan et al. (2022) | serious games; board games; anatomy; flow | Digital board game to rehearse and practice anatomy learning | High risk of bias |
| AntibioGame: A serious game for teaching medical students about antibiotic use | Tsopra et al. (2020) | serious games; antibiotics; infectious diseases; primary care; education; e-learning | AntibioGame is a case-based serious game to learn about use of antibiotics in primary care settings | High risk of bias |
| Applying an instructional design method to serious games - Experiences and lessons learned | Faber et al. (2018) | serious game; instructional design; education; emergency medicine | abcdeSIM is a digital serious game to prepare players in an emergency department by teaching the ABCDE method. | Not applicable |
| Appraisal of face and content validity of a serious game improving situational awareness in surgical training | Graafland et al. (2015) | / | Digital serious game to foster surgical residents' awareness towards equipment-related malfunctions in the operating room during minimally invasive surgery | Moderate risk of bias |
| A randomized controlled trial on teaching geriatric medical decision making and cost consciousness with the serious game GeriatriX | Lagro et al. (2014) | medical decision making; serious games; medical education; geriatric medicine | GeriatriX is a digital serious game for training knowledge about geriatrics content | High risk of bias |
| Beyond fun and games: Toward an adaptive and emergent learning platform for pre-med students with the UT TIME portal | Zielke et al. (2016) | serious games; gifted learners; online learning portal; asynchronous access; qualitative data; adaptive learning systems; emergent learning systems; blended learning | The Caregiver / UT TIME Portal is a digital serious game for students improving their knowledge about professionalism in social media use and about basic patient interviewing skills. | High risk of bias |
| Board game versus lecture-based seminar in the teaching of pharmacology of antimicrobial drugs - A randomized controlled trial | Karbownik et al. (2016) | basic medical pharmacology; antimicrobial drugs; medical education; board game | AntimicroGAME is a board game following the objective to teach the pharmacology of antimicrobial drugs. | Moderate risk of bias |
| Break out of the classroom: The use of escape rooms as an alternative teaching strategy in surgical education | Kinio et al. (2018) | escape room; gamification; active learning; CanMEDS; game-based learning | Analogue escape room for teaching preclerkship medical students about vascular surgery topics | High risk of bias |
| Comparative effectiveness of a serious game and an e-module to support patient safety knowledge and awareness | Dankbaar et al. (2017) | serious games; motivation; knowledge; performance; self-efficacy; design-based research; patient safety | Air-Medic-Sky-1 is a computer based serious game with the learning goals to "stimulate patient safety awareness and personal stress management" (p. 3) | High risk of bias |
| CureQuest: A digital game for new drug discovery | Chang et al. (2021) | game-based learning; drug discovery; translational science; team science; serious games; medical education | CureQuest is an adventure serious game to teach about drug discovery | Not applicable |
| Designing a serious game about critical appraisal of medical literature for pregraduate students | Mlika et al. (2020) | serious game; critical appraisal of medical literature; medical education | Digital serious game to help medical students learn how to critical appraise medical literature | Moderate risk of bias |
| Delivering a novel medical education "escape room" at a national scientific conference: First live, then pivoting to remote learning because of COVID-19 | Kaul et al. (2021) | development; education; escape room; gamification; game-based learning; interactive learning | Escape room for learning medical contents, especially pulmonary and critical care medicine, at a scientific conference | Not applicable |
| Development and evaluation of a serious game for teaching ICD-10 diagnosis coding to medical students. | Agudelo-Londono et al. (2019) | serious games; medical education; clinical coding; international classification of diseases; learning | CODIFICO is a digital serious game to train medical students in coding diagnosis according to ICD-10. | Moderate risk of bias |
| Escape into patient safety: Bringing human factors to life for medical students | Backhouse & Malik (2019) | / | Analogue escape room to learn about patient safety and principles of human factors in healthcare | Not applicable |
| Educational games in an obstetrics and gynecology core curriculum | O’Leary et al. (2005) | game theory in medical education; interactive learning of ectopic pregnancy | Boardgame in a "Jeopardy" style with "categories of epidemiology and differential diagnosis, risk factors, signs/symptoms, diagnosis, and treatment" (p.1849) | High risk of bias |
| Educational game development approach to a particular case: The donor's evaluation | Escribano et al. (2015) | / | Donor's Evaluation is a digital game-like simulation depicting the situation of evaluating potential organ donors as a hospital coordinator | Not applicable |
| Effectiveness of a serious game for medical education on insulin therapy: a pilot study | Diehl et al. (2015) | video games; medical education; insulin; diabetes mellitus; computer-assisted instruction | InsuOnLine is a browser based serious game to enhance learning about insulin administration (in primary care) | Low risk of bias |
| Efficacy of educational video game versus traditional educational apps at improving physician decision making in trauma triage: randomized controlled trial | Mohan et al. (2017) | / | The digital serious game app represents an emergency department helping residents improving their triaging skills. | High risk of bias |
| eMedOffice: A web-based collaborative serious game for teaching optimal design of a medical practice | Hannig et al. (2012) | computer-assisted instruction / methods; games; experimental; teaching / methods; education; medical; undergraduate / methods; user-computer interface | eMedOffice is a digital serious game that teaches how to set up a well-equipped medical practice | High risk of bias |
| Evaluation of Surgical Improvement of Clinical Knowledge Ops (SICKO), an interactive training platform | Nemirovsky et al. (2021) | medical education; surgical training; digital training; digital imaging; clinical management | Surgical Improvement of Clinical Knowledge Ops (SICKO) is a browser based serious game for fostering clinical decision-making in surgery | Not applicable |
| Exploring the influence of game design on learning and voluntary use in an online vascular anatomy study aid | Gauthier et al. (2015) | serious games; interactive learning environments; media in education; teaching/learning strategies; computer-mediated communication | Vascular Invaders is a web-based serious game, containing multiple game elements, designed for learning vascular anatomy in first-year medical students. | High risk of bias |
| Feeling the flow with a serious game workshop: GridlockED as Medical Education 2 study (GAME2 study) | Hale et al. (2021) | medical education; program evaluation; serious games | GridlockED is a analogue board game for learning about the patient flow in an emergency department | High risk of bias |
| Game-based e-learning is more effective than a conventional instructional method: A randomized controlled trial with third-year medical students | Boeker et al. (2013) | / | Uro-Island is an electronic adventure game to learn about urinalysis. | High risk of bias |
| Game-based learning for health professionals working in cancer care | Rodrigues et al. (2020) | game-based learning; health professionals; cancer care; side effects; health maintenance; unlimited input database | Digital serious game for learning "how to act efficiently when side effects occur during or after cancer treatment" (p.2) | Not applicable |
| Game-based training improves the surgeon's situational awareness in the operation room: A randomized controlled trial | Graafland et al. (2017) | videogame; medical education; smartphone; minimally invasive surgery; eHealth; cholecystectomy | Dr. Game, Surgeon Trouble® is a digital serious game in form of an arcade-type animated game to teach important surgical content and to prepare for adverse events in the surgical theatre. | High risk of bias |
| Game-based versus traditional case-based learning - Comparing effectiveness in stroke continuing medical education | Telner et al. (2010) | / | A board game based on the game "Snakes and Ladders" to enhance learning on stroke prevention and management. | High risk of bias |
| Gut Games: a board game to integrate basic and clinical sciences for the classroom | Katrikh et al. (2021) | flipped classroom; small group; game-based activity; medical education | GutGames is a board game serious game for medical students to learn about gastrointestinal diseases | Not applicable |
| Impact of using a 3D visual metaphor serious game to teach history-taking content to medical students: Longitudinal mixed methods pilot study | Alyami et al. (2019) | video games; instructional technology; memory; retention; metaphor; learning; clinical competence | Metaphoria is a 3D serious game equipped with visual metaphors aiming to support learning on history taking. | Low risk of bias |
| InsuOnline, an electronic game for medical education on insulin therapy: A randomized controlled trial with primary care physicians | Diehl et al. (2017) | diabetes mellitus; insulin; video games; medical education; continuing medical education; educational technology | InsuOnLine is a computer based serious game to enhance learning about insulin administration (in primary care) | High risk of bias |
| InsuOnline, a serious game to teach insulin therapy to primary care physicians: Design of the game and a randomized controlled trial for educational validation | Diehl et al. (2013) | diabetes mellitus; insulin; video games; medical education; educational technology; continuing medical education | InsuOnline is a digital adventure serious game for teaching primary care physicians about diabetes mellitus and dispensing insulin | Not applicable |
| L&D in the ED: A game-based approach to learning high-risk obstetric emergencies | Silverio et al. (2019) | emergency medicine; game; obstetrics; labor and delivery; precipitous delivery; board game | Board game for learning about labor, normal delivery, and delivery in emergency medicine contexts | Not applicable |
| Learning blood management in orthopedic surgery through gameplay | Qin et al. (2010) | / | Digital serious game for training blood management in orthopedic surgeries | High risk of bias |
| NEOGAMES: A serious computer game that improves long-term knowledge retention of neonatal resuscitation in undergraduate medical students | Hu et al. (2021) | serious games; neonatal resuscitation; knowledge retention; medical education; simulation-based education | NEOGAMES is a digital serious game for training undergraduate medical students in neonatal resuscitation. | Low risk of bias |
| Pedagotchi: Entwicklung einer neuartigen Lernanwendung für die Pädiatrie | Schmidt & Grigull (2018) | medical education; serious games; clinical reasoning; case based learning | PedaGotchi is a digital app-based serious game to improve knowledge in pediatrics. | High risk of bias |
| Playing cards on asthma management: A new interactive method for knowledge transfer to primary care physicians | Boulet et al. (2007) | asthma; game-based learning; knowledge implementation; medical education | "I suffer from asthma" is an interactive card game supporting the learning of asthma guidelines for primary care treatment, which is played in teams in a broader group. | High risk of bias |
| Preparing residents effectively in emergency skills training with a serious game | Dankbaar et al. (2017) | serious game; game-based simulation; emergency care training; clinical skills training; motivation | The simulation game abcdeSIM is used for family-residents helping them to prepare for their placement in an emergency department. | Moderate risk of bias |
| Ready patient one: How to turn an in-person critical care simulation scenario into an online serious game | Donovan et al. (2021) | healthcare education; Covid-19; serious game; escape room; choose your own adventure; cyoa; simulation in medical education | Serious game based on CYOA (create your own adventure) and Escape room concepts fostering knowledge about critical care concept (applying basic science principles to medical decision-making). | Not applicable |
| Serious games in formal medical education: An experimental study | Ribeiro et al. (2013) | / | Critical Transport is a digital serious game designed for undergraduate medical students to learn about the transport of critically ill patients. | High risk of bias |
| Serious games may improve physician heuristics in trauma triage | Mohan et al. (2018) | heuristics; triage; serious games; decision making; judgment | Two games, an adventure game and a puzzle game for learning about triaging. | High risk of bias |
| Serious game for teaching undergraduate medical students in cleft lip and palate treatment protocol | Palee et al. (2020) | game-based learning; serious game; cleft lip and palate | Cleft Island is a digital action-adventure serious game for medical students about cleft lip with or without cleft palate. | Moderate risk of bias |
| Serious game versus online course for pretraining medical students before a simulation-based mastery learning course on cardiopulmonary resuscitation - A randomized controlled study | Drummond et al. (2017) | / | The digital serious game "Staying Alive" is a point-and-click game that conveys learning content on the topic of cardiac arrest. | High risk of bias |
| Serious gaming for orthotopic liver transplant anesthesiology: A randomized control trial | Katz et al. (2017) | / | OLT Trainer is a serious game for training orthotopic liver transplantation | High risk of bias |
| The co-design, implementation and evaluation of a serious board game ‘PlayDecide patient safety’ to educate junior doctors about patient safety and the importance of reporting safety concerns | Ward et al. (2019) | medical education; junior doctors; embedded learning; serious game; patient safety; safety culture | PlayDecide Patient Safety is a serious board game to learn about patient safety, the importance of reporting safety concerns, and how to sharpen a culture of responsiveness from senior medical staff | High risk of bias |
| The effectiveness of a serious game versus online lectures for improving medical students’ coronavirus disease 2019 knowledge | Hu et al. (2021) | serious game; medical student; novel coronavirus disease | The digital game contains three missions for medical students to learn about COVID-19 and its requirements. | Low risk of bias |
| The impact of specially designed digital games-based learning in undergraduate pathology and medical education | Kanthan et al. (2011) | / | The two games Path to Success and The Path is Right are designed for medical education but not necessarily for pathology | High risk of bias |
| Use of a web-based game to teach pediatric content to medical students | Sward (2008) | active learning; informatics; medical education; web-based game | A web-based serious game based on the Pediatric Board Game used for teaching pediatric content to medical students | High risk of bias |
| User assessment of “InsuOnLine,” a game to fight clinical inertia in diabetes: A pilot study. | Diehl et al. (2015) | / | InsuOnLine is a serious game designed for primary care physicians to learn how to adequately prescribe insulin therapy | Not applicable |

*Note.* All studies listed in this table refer to populations in medical education.

| Title | First author & Year of publication | Keywords | Serious game | Risk of bias assessment |
| --- | --- | --- | --- | --- |
| A collaborative escape room as gamification strategy to increase learning motivation and develop curricular skills of occupational therapy students | Dugnol-Menéndez et al. (2021) | occupational therapy; education; escape room; gamification; students; collaborative; teamwork | Escape room game use for the education of occupational therapy students | High risk of bias |
| A jeopardy-style review game using team clickers | Cusick (2016) | active learning; problem-based learning; jeopardy review game; team clickers; student engagement | Jeopardy like quiz show for the classroom. | Not applicable |
| A preliminary study on the design of mobile educational game applied to pharmacology teaching | Shi et al. (2020) | educational games; pharmacology; RETAIN model; game design | An educational game relying on the RETAIN model for teaching pharmacy students about pharmacology | Not applicable |
| A serious game for patients' rights education | De Oliveira et al. (2021) | patients rights; serious game; serious games design framework; health professional education; higher education; health law | The PRITS serious game is a digital serious game to teach workers in medicine or healthcare about patients' rights and related laws in Switzerland. | Not applicable |
| A serious game for teaching nursing students clinical reasoning and decision-making skills | Johnsen et al. (2016) | community health nursing; computer simulation; education; problem-based learning; serious games; user-computer interface | Video-based serious game to prepare nurses for home healthcare with a particular focus on COPD | Not applicable |
| Co-designing a hybrid game for training use of proper personal protective equipment in different clinical scenarios | Merilampi et al. (2021) | co-design; game-based learning; hybrid game; infection control; nursing education; occupational safety; patient safety; passive RFID; personal protective equipment | Hybrid serious game to teach about the use of proper personal equipment in different scenarios for nursing students during COVID-19 | Not applicable |
| Comparison of the effects of virtual training by serious game and lecture on operating room novices’ knowledge and performance about surgical instruments setup: A multi-center, two-arm study | Akbari et al. (2022) | lecture; perioperative nursing; serious game; surgical instruments | PlaSurIn is a digital serious game to teach the right preparation of surgical instruments to operating room technology students | High risk of bias |
| Comparing the effects on learning outcomes of tablet-based and virtual reality-based serious gaming modules for basic life support training: Randomized trial | Aksoy (2019) | serious gaming; virtual reality; health care education | The 3DMedSim tablet-based serious game a dynamic learning environment for basic life support training. | High risk of bias |
| Designing and evaluating game-based learning for continuing pharmacy education using an "escape room" activity | Cole & Ruble (2021) | escape room; active learning; continuing education; pharmacy; pharmacy technicians | Escape room for continuous learning on medication errors for pharmacists and pharmacy technicians | High risk of bias |
| Designing and evaluating the effectiveness of a serious game for safe administration of blood transfusion: A randomized controlled trial | Tan et al. (2017) | nursing education; serious game; blood transfusion; patient safety; simulation | Digital serious game for improving knowledge and confidence in blood transfusion for nursing students | High risk of bias |
| Development and evaluation of a serious game to support learning among pharmacy and nursing students | Kayyali et al. (2021) | educational game; serious game; game design; online education; pharmacy education; information skills; nursing education | DOSE is a browser based serious game for increasing students confidence and use of the British National Formulary | High risk of bias |
| Development and questionnaire-based evaluation of virtual dental clinic: A serious game for training dental students | Wu et al. (2021) | clerkship; dental education; family dentistry; serious game; virtual dental clinic | Virtual Dental Clinic is a digital serious game for training clinical reasoning skills in dental students and enhancing cognitive knowledge in dentistry fields. | Moderate risk of bias |
| Escape-cardio: Gamification in cardiovascular physiotherapy. An observational study. | Ferrer-Sargues et al. (2021) | simulation; gamification; game-based learning; physiotherapy; escape rooms; cardiovascular | Escape-Cardio is an analogue escape room providing a learning opportunity for cardiovascular content | High risk of bias |
| Escaping the professional silo: An escape room implemented in an interprofessional education curriculum. | Friedrich et al. (2018) | interprofessional education; students; communication; escape room; teamwork; evaluation | Analogue escape room promoting interprofessional communication and teamwork | High risk of bias |
| Educational game application development on classification of diseases and related health problems treatment in android platform | Sunindya et al. (2017) | game; KKPMT (Klasifikasi dan Kodifikasi Penyakit dan Masalah Terkait); android | Digital serious game for improving the skill of coding a disease | High risk of bias |
| Educational gaming for dental students: Design and assessment of a pilot endodontic-themed escape game | Aubeux et al. (2020) | educational game; endodontics; escape room; serious gaming; simulation; teamwork | Analogue escape room for dental students learning about pulpal and periapical diseases | Moderate risk of bias |
| Engaged in learning neurorehabilitation: Development and validation of a serious game with user-centered design | Savazzi et al. (2018) | / | Digital serious game for physiotherapists to train treatment of patients in neurorehabilitation | High risk of bias |
| Evaluation of a 3D serious game for advanced life support retraining | Buttussi et al. (2013) | education; retraining; advanced cardiac life support; computer simulation; serious gaming; evaluation studies | EMSAVE is a 3D scenario-based serious game to refresh ALS knowledge and decision-making skills. | Moderate risk of bias |
| From experiencing to critical thinking: a contextual game‐based learning approach to improving nursing students’ performance in electrocardiogram training | Chang et al. (2020) | digital game-based learning; contextual learning; critical thinking; nursing education | A digital serious game to enhance nurses understanding of ECG waves. | Moderate risk of bias |
| Gamification in cardiovascular pharmacology course as real work simulation by case on medical sciences | Saeidmirzaei et al. (2020) | pharmacology course; gamification; motivation; medical education; active learning | Digital serious game with pharmacological learning objectives for nursing and paramedic students. | High risk of bias |
| Healthcare management through organizational simulation | Basole et al. (2013) | healthcare management; organizational simulation; serious games; health advisor; information complexity; decision-making | Health Advisor is a digital serious game for learning how to manage several clients through the US American healthcare system | High risk of bias |
| Improving nursing students' COVID-19 knowledge using a serious game | Hu et al. (2022) | COVID-19 education; education; knowledge retention; nursing students; serious game | The COVID-game is a digital serious game to support nurses online learning about COVID-19 and it's requirements. | Low risk of bias |
| Nursing students’ perceptions of a video-based serious game’s educational value: A pilot study | Johnsen et al. (2018) | clinical decision-making; computer simulation; e-learning; games; health care; survey; validation | Prototype of a digital video-based serious game covering COPD topics to prepare nurses for placements in home healthcare or hospital settings. | Moderate risk of bias |
| Nursing students’ perceptions of combining hands-on simulation with simulated patients and a serious game in preparing for clinical placement in home healthcare: A qualitative study | Johnsen et al. (2021) | blended learning; home healthcare; nursing education; serious game; simulation | Digital video-based serious game to prepare nursing students for home healthcare operations | Moderate risk of bias |
| Teaching clinical reasoning and decision-making skills to nursing students: Design, development, and usability evaluation of a serious game. | Johnsen et al. (2016) | clinical decision-making; community health nursing; computer simulation; education; problem-based learning; user-computer interface | "I cannot breathe" is a video-based simulation game to train nurses' clinical reasoning and decision-making skills when caring for COPD patients in home healthcare settings. | Moderate risk of bias |
| The effects of students' learning anxiety and motivation on the learning achievement in the activity theory based gamified learning environment | Su (2017) | mobile game-based learning; activity theory; ARCS model; cardiac catheterization | 3D-CCGBLS (3D Cardiac Catheterization Game-Based Learning System) is a digital game offering a learning assessment for cardiac catheterization. | High risk of bias |
| The effectiveness of serious games designed for infection prevention and promotion of safe behaviors of senior nursing students during the COVID-19 pandemic | Calik et al. (2022) | serious game; nursing student; education; COVID-19; knowledge; skill competency | The proposed digital serious game had the learning objective to teach COVID-19 knowledge to nursing students | High risk of bias |
| Three-dimensional game-based cardiopulmonary bypass training | Bonet et al. (2021) | nursing education; cardiopulmonary bypass training; serious games; game-based learning; simulation; practical skills; social skills | Virtual Perfusionist is a 3D serious simulation game to train nurses in management of cardiopulmonary bypass machines | Not applicable |
| VIMED: Fish-tank approach to nurse practical training | Barr et al. (2008) | / | VI-MED is a virtual environment simulating a nurse's shift in a hospital ward | Not applicable |

*Note.* All studies listed in this table refer to populations in the education of healthcare professionals.

| Title | First author & year of publication | Keywords | Serious game | Risk of bias assessment |
| --- | --- | --- | --- | --- |
| A didactic escape game for emergency medicine aimed at learning to work as a team and making diagnoses: Methodology for game development | Abensur Vuillaume et al. (2021) | training techniques; educational technique; game theories; emergency medicine; games; education; escape game; simulation-based training; pedagogical; serious games; emergency medicine training | Escape game for promoting team work abilities of emergency medical teams | Not applicable |
| Co-designing a serious game to train emergency medical services | El Mawas & Cahier (2013) | emergency crisis management; serious games; crisis training; discussion forum; participative design | Serious game to teach persons working in emergency medical services knowledge and skills necessary to be part of emergency medical teams | Not applicable |
| Creating GridlockED: A serious game for teaching about multipatient environments | Tsoy et al. (2019) | / | GridlockED is a collaborative serious game that teaches players how to handle and manage multiple patients during an eight-hour ED shift (i.e. multipatient environment). | Not applicable |
| Escape the simulation room | Sanders et al. (2021) | escape room; health care; teamwork; communication; emergency medicine | Analogue escape room mimicking interdisciplinary teamwork environments in a pediatric setting | Not applicable |
| Operationalizing resilient healthcare concepts through a serious video game for clinicians | Jackson et al. (2020) | resilience; safety II; serious video game; healthcare; resilience engineering; gamification; resilient healthcare; serious games; safety; feasibility; reflection; survey | Resilience Challenge is a digital serious game that teaches organizational resilience for healthcare workers. | Moderate risk of bias |
| Prospective cohort study on surgeons’ response to equipment failure in the laparoscopic environment | Graafland et al. (2014) | / | The serious game included 37 problem scenarios on equipment malfunction in minimally invasive surgery situations. | High risk of bias |
| Project sanitarium: Playing tuberculosis to its end game | Donald et al. (2017) | serious games; games with purpose; games for change; games education; educational games; interdisciplinary working; collaborative research | Project Sanatorium is a serious game designed for learning about the treatment of tuberculosis by means of presenting data from recent clinical trials. | Not applicable |
| Serious gaming technology in major incident triage training: A pragmatic controlled trial | Knight et al. (2010) | / | Triage Trainer is a digital serious game facilitating the learning of triaging in major / mass incident situations. | High risk of bias |
| The influence of a serious game’s narrative on students’ attitudes and learning experiences regarding delirium: An interview study | Buijs-Spanjers et al. (2020) | delirium; education; serious game; narrative | The Delirium Experience is a video based serious game for teaching the delirium syndrome to a broader audience | Low risk of bias |

*Note.* All studies listed in this table refer to populations in the education of both medical and healthcare professionals.
